# A combined cholecystoduodenocolic and gastrocolic fistula with a common orifice in the colon: A case report

**DOI:** 10.1016/j.ijscr.2025.111899

**Published:** 2025-09-04

**Authors:** Bahar Amiri, Asadollah Roshani, Mehdi Karimi, Arian Karimi Rouzbahani, Behzad Yaghoubi

**Affiliations:** a Student Research Committee, Lorestan University of Medical Sciences, Khorramabad, Iran; b Department of Surgery, Lorestan University of Medical Sciences, Khorramabad, Iran; c Faculty of Medicine, Bogomolets National Medical University (NMU), Kyiv, Ukraine

**Keywords:** Biliary fistula, Case report, Cholecystoduodenocolic, Gallstone disease, Gastrocolic

## Abstract

**Introduction:**

Gallbladder fistulas primarily connect to the duodenum (up to 83.3 % of cases) or colon (up to 24.5 %), with rare connections to other gastrointestinal organs. This report documents the clinical, pathological, and laboratory features and the management of a unique case involving a combined cholecystoduodenocolic and gastrocolic fistula with a common orifice in the colon.

**Presentation of the case:**

A 46-year-old Iranian male with methadone addiction was hospitalized on December 12, 2024, due to abdominal pain, significant weight loss, and severe diarrhea with melena, who had delayed seeking medical attention because methadone alleviated his symptoms.

**Discussion:**

Biliary fistulae can involve multiple organs and often present with non-specific symptoms, complicating diagnosis. Imaging revealed severely hypoechoic tissue in the gallbladder, suggesting a fistula. Upper endoscopy showed only a duodenal ulcer fistulized to the transverse colon, allowing the endoscope probe to be easily inserted into the colon without pressure. The surgical approach included cholecystectomy, resection and anastomosis of the involved colon, antrectomy, and removal of a portion of the proximal duodenum (D1), followed by a Billroth II reconstruction.

**Conclusion:**

Simultaneous fistulization involving the gallbladder, stomach, duodenum, and colon is possible, though rare. This case underscores the importance of thorough individualized surgical approaches to optimize outcomes in similar cases.

## Highlights

• Biliary fistulas are rare complications of gallstone disease.
• The most common types are cholecystoduodenal fistulas.
• Biliary fistulae can involve multiple organs simultaneously, including cholecystoduodenocolic and gastrocolic fistulas.

## 1 Introduction

Biliary fistulas are rare complications of gallstone disease, occurring in 3–8 % of cases ([1], [2], [3]). The most common types are cholecystoduodenal, cholecystocolonic, and cholecystogastric fistulas (4). Gallstone disease is consistently recognized as the primary underlying condition, with patients typically aged 54.5 to 81.5 years and a predominance of females (55 %–66 %) ([5], [6], [7]). The mechanism underlying cholecystoenteric fistulae is well understood. Large gallstones (especially those that are large or impacted with stones) compress the gallbladder wall, causing wall ischemia, necrosis, erosion, and fistula formation with the adjacent viscus (8). These fistulas often present with non-specific symptoms, complicating preoperative diagnosis, which may occur incidentally during endoscopy or imaging ([2], [3], [4]). Diagnosis usually occurs intraoperatively or through advanced imaging techniques (9). Pneumobilia can be a presenting sign in some cases (10). Treatment varies, including open surgery, laparoscopic techniques, and endoscopic interventions (9,11). Traditionally, open cholecystectomy was the standard treatment, but laparoscopic and endoscopic approaches have shown favorable results (3). Early recognition and management are crucial to prevent serious complications like peritonitis and septic shock (9). These fistulae can involve multiple organs simultaneously, as demonstrated by cases of combined cholecystoduodenal fistulas with cholecystocolonic fistulas (10,12,13), or cholecystogastric fistulas with cholecystocolonic fistulas (14).

In this particular case, we thoroughly reported the clinical, pathological, laboratory features, and management of a 46-year-old man who had biliary fistulas. This is the initial documented case of a combined cholecystoduodenocolic and gastrocolic fistula with a common orifice in the colon.

## 2 Presentation of the case

A 46-year-old male Iranian patient with a history of methadone addiction was hospitalized on December 12, 2024, due to complaints of diarrhea and abdominal pain. The abdominal pain was generalized but predominantly located in the epigastric region. It had started four months before hospitalization, occurring in seven episodes, and improved with methadone, which led the patient to delay seeking medical attention. The pain worsened with eating and was alleviated by methadone; it was not positional. The patient also reported anorexia and non-biliary vomiting that contained food particles, along with a significant weight loss of 15 kg over the past 40 days.

In the two weeks preceding hospitalization, abdominal pain decreased, but he experienced severe diarrhea, characterized by melena. As the disease progressed, he noted that food was excreted in the stool in an undigested form, without any alteration in shape, shortly after eating. The patient had no history of other diseases or notable medical conditions, aside from his methadone addiction, for which he took 40 mg daily. He also had a smoking history of 30 pack-years.

Upon examination, his vital signs revealed a pulse rate of 123 beats per minute, blood pressure of 100/70 mmHg, and a respiratory rate of 27 breaths per minute. The abdominal examination revealed a soft abdomen with generalized tenderness, but no rebound tenderness or guarding was noted. Other physical examinations were unremarkable.

## 3 Diagnostic assessment

Laboratory assessments included complete blood count (CBC), blood biochemistry, coagulation, and stool exam. The patient presented with severe anemia, characterized by low levels of RBC, hemoglobin, and hematocrit, along with low MCV, MCH, and MCHC. The WBC and platelet count were also elevated, suggesting a reactive process. Inflammatory markers were notably high, with a positive CRP and an elevated ESR, indicating an ongoing inflammatory response. Furthermore, biochemistry results revealed low sodium, potassium, calcium, TIBC, and iron levels, as well as low serum albumin, pointing to possible nutritional deficiencies. Additionally, the stool test results were normal. A summary of these findings is provided in Table 1.

**Table 1:** Laboratory tests.

Test	Unit	Normal range	Before treatment	After treatment
Blood count				
RBC	10^12^/L	4.5–5.8	3.18	–
Hemoglobulin	g/dl	14–17	6.9	9.6
Hct	%	41–50	22.8	30.5
MCV	F lit	80–100	77	–
MCH	pg	27–33	23	–
MCHC	g/dl	31–36	29.8	–
WBC	10^9^/L	4.0–10.0	10.9	–
Platelet	10^3^/ μl	150–450	676	–
RDW-CV	%	11.0–14.5	17.4	–

Coagulation				
PT	s	11–15	14.1	13.2
PTT	s	20–40	20	–
INR	s	0.76–1.45	1.28	1.18

Biochemistry				
CRP	Qualitative	Negative	Positive (++)	–
ESR	mm/h	0–22	56	–
FBS	mg/dL	70–100	89	–
BUN	mg/dL	8–20	37	–
Cr	mg/dL	0.9–1.3	0.94	–
Na^+^	mmol/L	135–145	132.5	–
PK^+^	mmol/L	3.5–5.0	3.31	–
Ca^2+^	mg/dL	8.6–10.8	7	–
AST	U/L	<37	24	–
ALT	U/L	<41	14	–
ALP	U/L	80–335	198	–
Total Bilirubin	mg/dL	0.1–1.2	0.17	–
Direct Bilirubin	mg/dL	<0.2	0.12	–
Albumin, Serum	g/dL	3.5–5.2	2.3	–
Amylase	IU/L	0–100	18	–
Lipase	IU/L	<60	5	–
TIBC	mg/dL	230–440	188	–
Fe	μg/dL	65–175	24	–

[Abbreviations: Red Blood Cells (RBC); White Blood Cells (WBC); Hematocrit (Hct); Mean corpuscular volume (MCV); Mean corpuscular Hemoglobin (MCH); mean corpuscular hemoglobin concentration (MCHC); Prothrombin Time (PT); Partial Thromboplastin Time (PTT); International Normalized Ratio (INR); Erythrocyte sedimentation rate (ESR); C-reactive protein (CRP); Blood urea nitrogen (BUN); Sodium (Na^+^); Potassium (K^+^); Calcium (Ca^2+^); Aspartate aminotransferase (AST); alanine aminotransferase (ALT); Alkaline phosphatase (ALP); Fasting blood sugar (FBS); Total iron binding capacity (TIBC)].

**Fig. 1:**
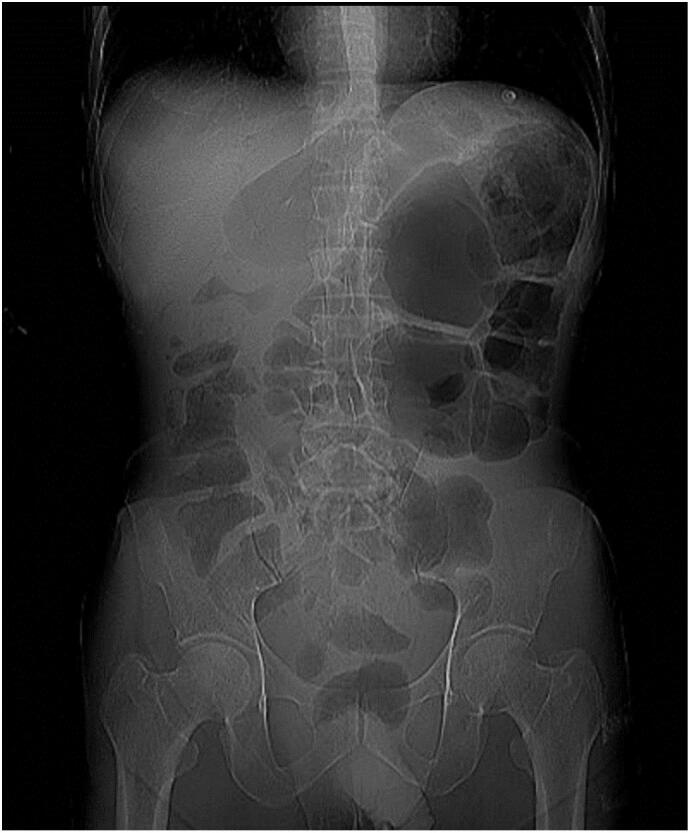
On the plain abdominal X-ray alone, there was no definitive visible evidence of a cholecystoduodenocolic or gastrocolic fistula.

An abdominal ultrasound was performed, revealing severely hypoechoic tissue with an air shadow in the gallbladder region, suggesting the possibility of a gallbladder fistula. A CT scan and a standing abdominal X-ray were performed to evaluate the abdominal problems further. In the abdominal X-ray, the bowel gas pattern appeared somewhat distended, especially in the right upper quadrant. Still, there was no clear evidence of air in the biliary tree (no obvious pneumobilia). Also, no obvious abnormal gas was collected outside the bowel lumen (Fig. 1).

**Fig. 2:**
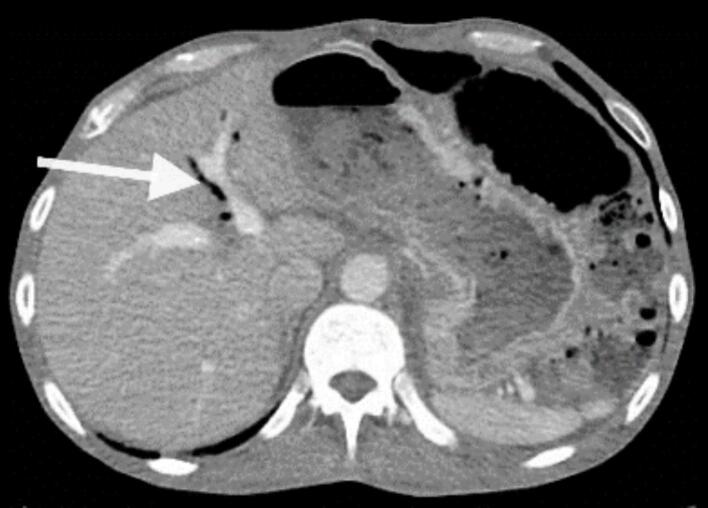
The white arrow points to the presence of air (black spots) within the biliary tree or gallbladder, which is abnormal and suggests pneumonia. This finding highly suggests a fistulous connection between the gallbladder and adjacent bowel (likely duodenum or colon).

The CT image shows pneumobilia with air in the gallbladder region, indicating an abnormal connection between the gallbladder and the adjacent bowel (Fig. 2).

Upper endoscopy revealed a duodenal ulcer fistulized to the transverse colon, measuring about 40 × 40 mm. This allowed the endoscope probe to be easily inserted into the colon without pressure.

## 4 Therapeutic intervention

The patient's nutrition status improved through TPN, and hydration and volume correction were performed by administering normal saline and a three-unit transfusion of packed red blood cells. On December 16, 2024, the patient underwent surgery, which involved cholecystectomy, resection and anastomosis of the involved colon, antrectomy, and removal of a portion of the proximal duodenum (D1), followed by a Billroth II reconstruction (a gastro-jejunostomy of the remaining stomach to the first jejunal loop).

**Fig. 3:**
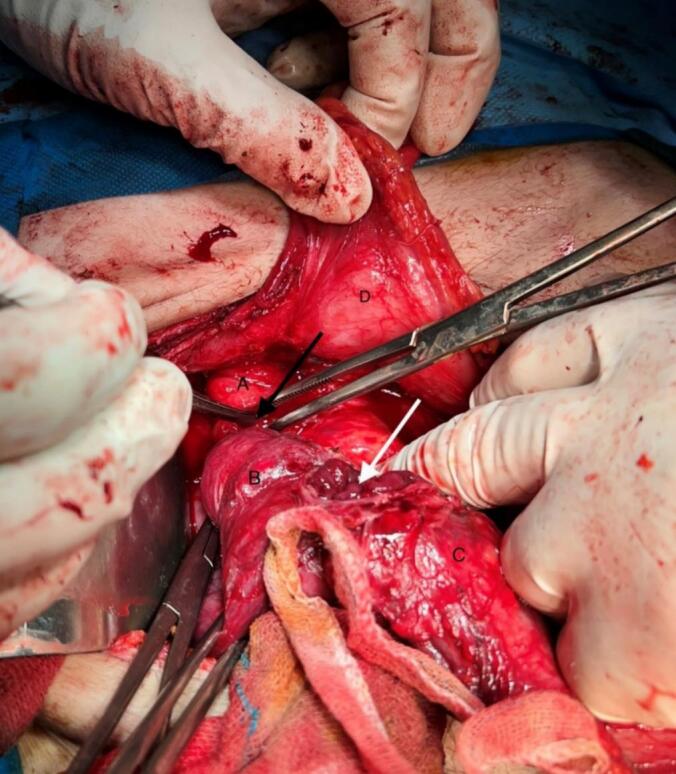
Intraoperative image. The black arrow indicates the fistula from the gallbladder (letter A) to the duodenum (letter B), and the white arrow indicates the hole on the transverse colon (letter C) separated from the stomach (letter D) and duodenum.

After prep and drape under general anesthesia in the supine position, a midline incision was made, and the abdominal cavity was examined. Adhesion of the gallbladder to the duodenum, duodenum to the transverse colon, and transverse colon to the gastric antrum was observed. Adhesions were first released, and enterolysis was performed (Fig. 3).

**Fig. 4:**
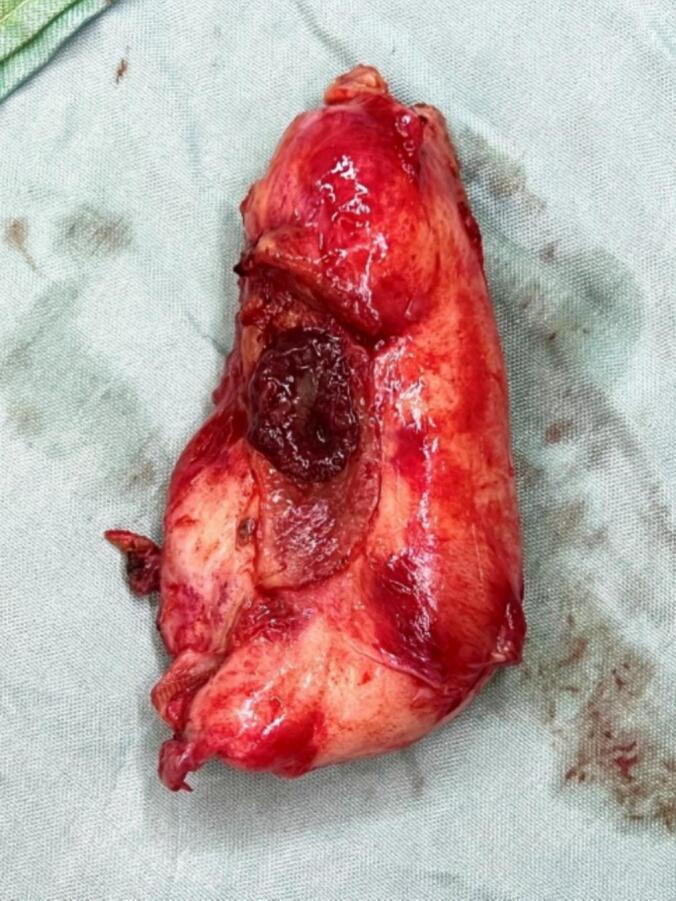
The gallbladder with a 2 cm hole in it removed during an open cholecystectomy.

**Fig. 5:**
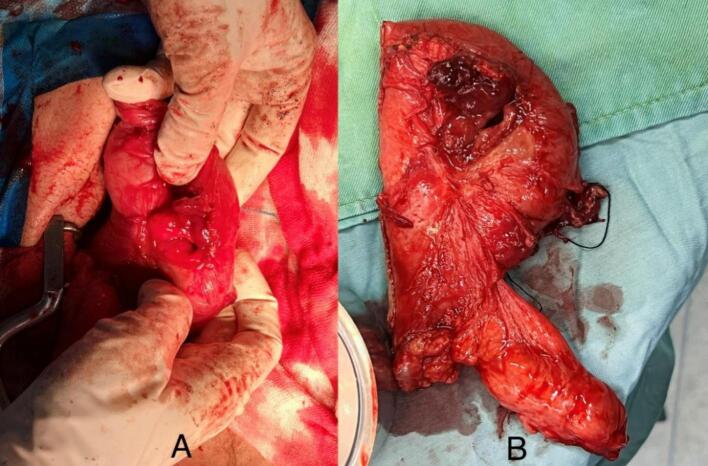
The Stomach and duodenum. (A) The posterior wall of the stomach antrum has a two cm hole; (B) The stomach antrum and the proximal portion of the duodenum (D1) have been cut and separated with a stapler.

**Fig. 6:**
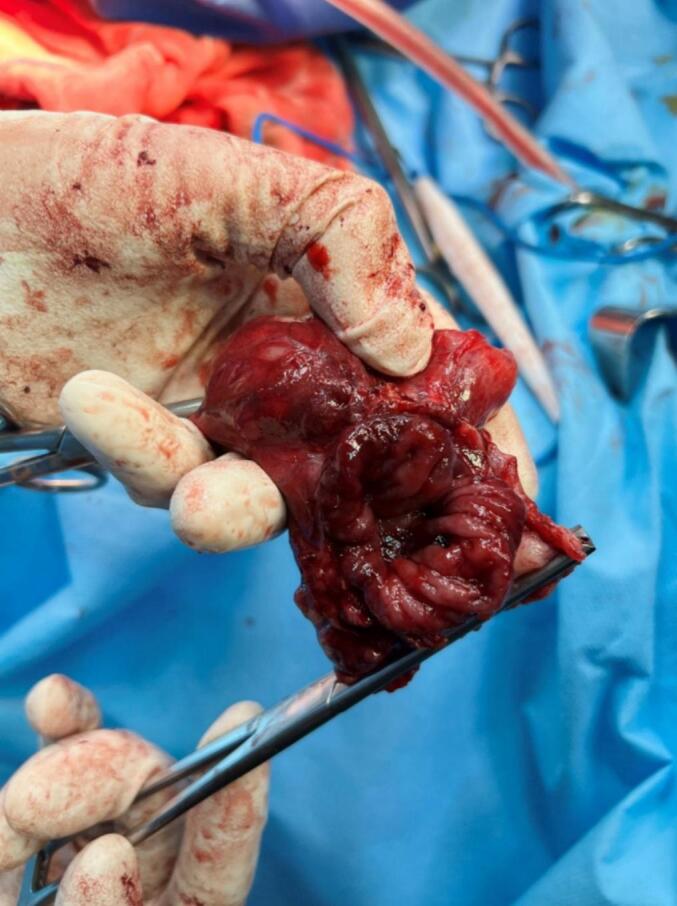
The transverse colon with a 4 cm hole in it.

The hepatic flexure of the colon was released, the gallbladder was separated from the duodenum, and the duodenum was co-frozen. One hole on the gallbladder (Fig. 4), a four-centimeter hole in the proximal portion of the duodenum (D1), a two-centimeter hole before the pylorus on the gastric antrum (Fig. 5), and a four-centimeter hole in the transverse colon were observed (Fig. 6).

The gallbladder was released from the liver bed with a cautery, and after exploration, the cystic duct and artery were double ligated and cut. After separating the colon from the duodenum and gastric antrum, the colon was resected in the perforated area, and anastomoses were performed end-to-end and in two layers.

Antrectomy and removal of the proximal portion of the duodenum (D1) was performed using two linear staplers and reinforced with a layer of Lembert sutures. After closing the duodenal stump the remaining portion of the duodenum, the jejunum, 45 cm from the ligament of Treitz, was elevated, and a gastrojejunostomy anastomosis or a Billroth II reconstruction was performed. The gallstone was evacuated. We did not identify any stones during pre-operative imaging or intraoperatively.

A loop ileostomy was placed 20 cm from the ileocecal valve in RLQ. After ensuring complete hemostasis and counting the number of gas and long gas, a drain was placed. The fascia was sewn with loop string, and the skin was repaired with nylon string. The ileostomy was sutured.

## 5 Follow-up and outcomes

After surgery, the patient remained nil per os (NPO) for 48 h. A delay in starting enteral nutrition is often due to severe nausea in the patient, which can lead to difficulties in tolerating nutrition and an increased risk of aspiration. Two units of packed red blood cells were transfused to the patient, and the patient's hemoglobin reached about 10 g/dl. Two days postoperatively, a liquid diet was initiated, which the patient tolerated well. Over the next 5 days, the diet was changed to regular, and the patient was discharged after tolerating PO and passing gas and stool. The drain was removed a few days postoperative day, with no evidence of postoperative drainage or complications.

The pathology report indicated only inflammation without any evidence of malignancy.

After three months of assessment in March 2025, a physical examination revealed no pain, with stable vital signs: Temperature 36.8 °C, blood pressure 120/78 mmHg, heart rate 100 bpm, respiratory rate 22 bpm, and oxygen saturation 99 % in room air. The abdomen was soft, without any tenderness. Other systematic examinations were unremarkable. The subsequent postoperative recovery was uneventful. Two months after the surgery, we performed an ileostomy reversal. We intend to perform periodic upper endoscopy to monitor the gastric stump for any potential malignant transformation in future follow-ups.

## 6 Timeline

In August 2024, the patient experienced abdominal pain, which decreased by December 1, 2024, leading to severe diarrhea with undigested food in the stool. Hospitalized on December 12, 2024, vital signs indicated tachycardia and hypotension. Hydration and volume correction were performed by administering normal saline and three packed red blood cell injections. An ultrasound on December 13, 2024, suggested a gallbladder fistula. After a CT scan on December 14, 2024, an endoscopy on December 15, 2024, revealed a duodenal ulcer fistulized to the colon. The patient underwent surgical intervention on December 16, 2024. (Table 2).

**Table 2:** Timeline.

Date	Event description
August 2024	The patient began experiencing generalized abdominal pain, primarily in the epigastric region. Pain occurred in seven episodes and improved with methadone, leading to delayed medical consultation.
December 1, 2024	The patient developed severe diarrhea characterized by melena. The abdominal pain was relieved, with undigested food being excreted in the stool shortly after eating.
December 12, 2024	The patient was hospitalized due to ongoing abdominal pain and diarrhea. Vital signs recorded: pulse rate, 123 bpm; blood pressure, 100/70 mmHg; respiratory rate, 27 breaths per minute. Abdominal examination revealed tenderness without rebound tenderness or guarding. Hydration and volume correction were performed by administering normal saline and three packed red blood cell injections.
December 13, 2024	Ultrasound was performed to assess the abdominal condition, revealing a severely hypoechoic tissue with air shadow in the gallbladder region, suggesting the possibility of a gallbladder fistula.
December 14, 2024	A CT scan and a standing abdominal X-ray were performed to evaluate the abdominal issues further.
December 15, 2024	Upper endoscopy was performed, revealing a duodenal ulcer that had fistulized to the colon.
December 16, 2024	The patient underwent surgical intervention, which involved cholecystectomy, resection and anastomosis of the involved colon, antrectomy, and removal of a portion of the proximal duodenum (D1), followed by a Billroth II reconstruction.
March 2025	The follow-up after surgery indicated that the postoperative recovery was uneventful.

## 7 Discussion

Cholecystoenteric fistulae are rare complications of gallstone disease, occurring in 0.5–0.9 % of cholecystectomies (10). Cholecystoduodenal fistulae are most common, followed by cholecystocolonic fistulae (12). Multiple fistulae can coexist in rare cases, such as combined cholecystoduodenal and cholecystocolonic fistulae (10,12). Despite advances in imaging techniques, preoperative diagnosis remains challenging, with many instances discovered incidentally during surgery. Diagnostic tools include ultrasonography, CT scans, ERCP, and MRI, which can provide detailed imaging of the biliary system. ERCP is particularly effective for both diagnosis and treatment, potentially reducing surgery costs, morbidity, and mortality (15). Computed tomography plays a crucial role in preoperative imaging; however, its effectiveness remains limited (16). Management typically involves surgical intervention, which may include laparoscopic cholecystectomy, subtotal cholecystectomy, and/or open repair of enteric fistulae (10,17). While open surgery remains the primary treatment method, laparoscopic approaches are also viable for some cases (18). Conservative treatment, including antibiotics and nutritional support, may also be employed (17). Due to the rarity and complexity of these cases, meticulous preoperative planning and surgical expertise are crucial for successful outcomes (10).

While existing literature addresses various combinations of biliary fistulas, a notable gap remains concerning the simultaneous occurrence of cholecystoduodenocolic and gastrocolic fistula in a single patient. This unique presentation presents distinct diagnostic and therapeutic challenges. This article aims to highlight this rare case and detail the complexities involved in its management, including cholecystectomy, resection and anastomosis of the involved colon, antrectomy, and removal of a portion of the proximal duodenum (D1), followed by a Billroth II reconstruction. We aim to provide insights highlighting the necessity of individualized strategies to manage such complex cases.

Several case reports document individual types of biliary fistulas or combinations of two.

Huang et al. (2022) discussed the surgical management of cholecystoenteric fistulas in a series of 29 cases, which included one notable case of a combined cholecystoduodenal and cholecystocolonic fistula. This case presented diagnostic challenges, as the patient exhibited symptoms consistent with both fistulas. Diagnosis was achieved through advanced imaging techniques such as CT and MRCP, which helped delineate the anatomy and the extent of the fistulous connections. The management involved a tailored surgical approach, including cholecystectomy and repair of the fistulae, emphasizing the importance of individualized treatment strategies based on the specific presentation of each patient (13). This study is one of the studies that resembles our case in terms of the open surgical management method employed.

Bohanon et al. (2024) presented a rare case of a combined cholecystocolonic and cholecystoduodenal fistula presenting with pneumobilia. Their case highlights diagnostic challenges, noting the importance of considering biliary fistulas in patients with atypical presentations. Diagnosis was achieved through imaging, and surgical management included cholecystectomy with fistula takedown and repair of the affected structures (10). Sidhu et al. (2024) reported a rare cholecysto-duodenocolonic fistula secondary to cholelithiasis. Their report underscores the importance of a high index of suspicion and thorough investigation in patients with gallstone disease presenting with unusual symptoms. The diagnostic method involved CT imaging, and the surgical approach included cholecystectomy, division of the fistula, and repair of the duodenum and colon (12).

Zhu et al. (2023) presented a case of laparoscopic management of combined cholecystogastric and cholecystocolonic fistulae, demonstrating the feasibility and safety of a minimally invasive approach in selected cases. Laparoscopic cholecystectomy and fistula division were performed with successful closure of the gastric and colonic defects (14). This case illustrates the potential for minimally invasive techniques to address complex fistulas, providing benefits such as reduced recovery time and postoperative pain.

## 8 Conclusion

While previous studies have documented various combinations of biliary fistulas, the simultaneous involvement and fistulisation of the four organs, including the gallbladder, stomach, duodenum, and colon in a patient, has not been previously reported. Furthermore, the extensive surgical approach, which involves cholecystectomy, resection and anastomosis of the involved colon, antrectomy, and removal of a portion of the proximal duodenum, followed by a Billroth II reconstruction, distinguishes our case from previously published reports, emphasizing the unique surgical challenges.

## Author contribution

B.A, A.R, A.KR, M.K and B.Y contributed to the design and implementation of the research and to the writing of the manuscript.

## Consent for publication

Written informed consent for publication of their clinical details and clinical images was obtained from the patient. A copy of the consent form is available for review by the Editor of this journal.

## Ethics approval and consent to participate

This case report did not require ethical approval from Ethics Committee in our institution. We have a written and signed consent to publish the information from the patient prior to submission. Our patient gave his consent for images and information about himself relating to the subject matter above to appear in the identified journal and associated publications.

## Guarantor

Behzad Yaghoubi.

## Patient perspective

The patient was satisfied with his treatment, and the subsequent postoperative recovery was uneventful.

## Funding

Not applicable.

## Declaration of competing interest

The authors declare that they have no competing interests.

## Data Availability

The datasets used during the current study are available from the corresponding author on reasonable request.
